# Neurosyphilis manifesting with unilateral visual loss and hyponatremia: a case report

**DOI:** 10.1186/1471-2334-11-17

**Published:** 2011-01-15

**Authors:** Katrin Milger, Vera Fleig, Anke Kohlenberg, Thomas Discher, Jürgen Lohmeyer

**Affiliations:** 1 Department of Internal Medicine II, Justus-Liebig University Giessen, Klinikstr. 36, 35392 Giessen, Germany; 2 Department of Medical Microbiology, Justus-Liebig University Giessen, Frankfurterstr. 107, 35392 Giessen, Germany

## Abstract

**Background:**

Syphilis is called the chameleon of the diseases due to its variety of its clinical presentations, potentially affecting every organ of the body. Incidence of this ancient disease is once again on the increase worldwide.

**Case presentation:**

We here report an unusual case of neurosyphilis manifesting with unilateral visual loss and hyponatremia. The patient also had primary syphilitic lesions and was concomitantly diagnosed with Human Immunodeficiency Virus (HIV), Hepatitis B Virus (HBV) and Hepatitis C Virus (HCV) infection. Treatment with ceftriaxone and prednisolone, completely resolved the hyponatremia and visual acuity was partially restored.

**Conclusion:**

Awareness of syphilis as a differential diagnosis is important as previously unreported presentations of neurosyphilis can arise, especially in HIV infected patients.

## Background

Incidence of syphilis is once again increasing throughout the world [1]. It predominantly spreads among gay men and other "Men who have Sex with Men" (MSM), in whom Human Immunodeficiency Virus (HIV) co-infection is often present.

The disease is known as the great mimicker because of its wide range of clinical presentations.

Manifestations have been described in almost every organ making diagnosis difficult. Neurosyphilis, the syphilitic infection of the nervous system (NS), has been known and studied for more than a century [2,3]. It is caused by dissemination of *Treponema pallidum *to the cerebrospinal fluid (CSF) and meninges [4]. This neuroinvasion occurs in early syphilis. Symptomatic neurosyphilis can develop at any stage of the disease [5].

## Case presentation

**Figure 1:**
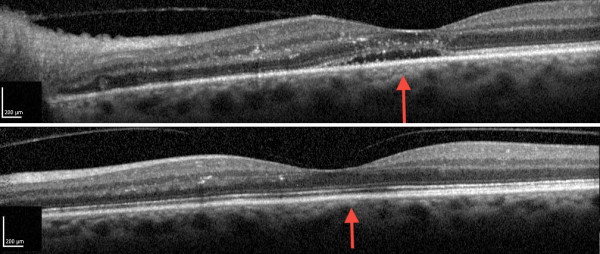
**Optical coherence tomography (OCT) image of the left eye**. Upper image: optic disk swelling (red arrow) at presentation. Lower image: Red arrow points to the former optic disk swelling that is resolved after three weeks of treatment.

**Figure 2:**
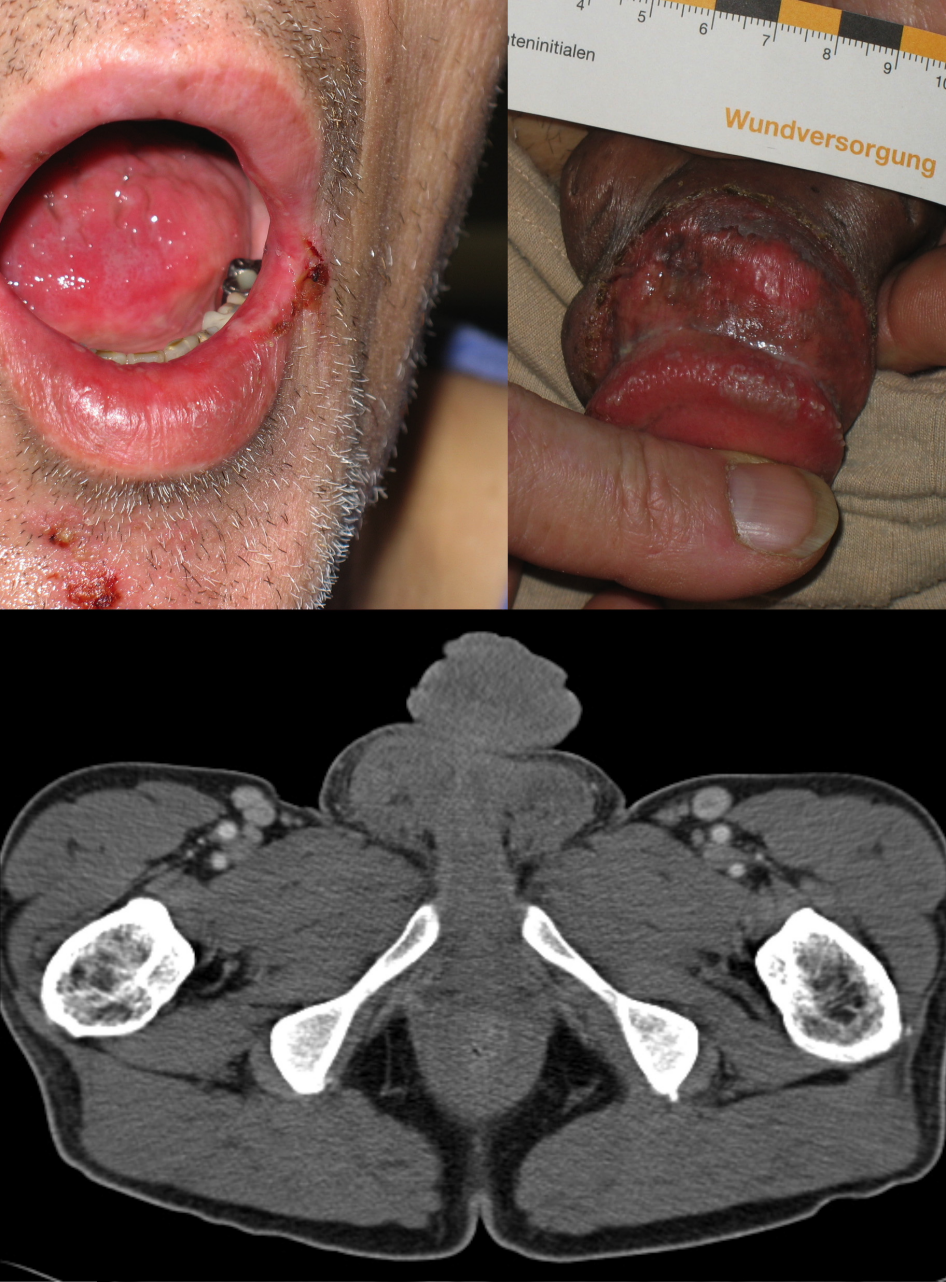
**Syphilitic lesions at presentation**. Upper left image: Perioral syphilitic lesion. Upper right image: Genital syphilitic lesion. PCR of smears confirmed presence of *Treponema pallidum *in both locations. Lower Image: CT scan: marked genital and scrotal oedema and inguinal lymphadenopathy.

A 48-year-old German Caucasian truck driver presented to the ophthalmologist with unilateral visual loss. Upon eye examination, visual acuity was 0.8 in the right eye and 0.02 in the left eye (normal visual acuity = 1). Fundoscopic examination showed swelling of the optic disk and serous retinal detachment in the left eye (Figure 1, upper image). Slit lamp exam did not reveal any additional pathology. Therefore, the diagnosis of unilateral papillitis was retained. Additionally, oral ulcers and thrush were identified (Figure 2, upper left image), hence an HIV test was ordered. HIV-serology was positive and the patient was referred to the infectious disease department of the University Hospital of Giessen.

Regarding his medical history, the patient had been asthenic and anorectic for 7 weeks and had lost 12 kg. He received a course of oral antibiotics (3 days of azithromycin) three weeks prior to presentation due to sinusitis. He had a burning pain when swallowing, but no other complaints. Apart from sinusitis no pre-existing medical conditions were known. The risk factor for HIV infection was MSM contacts.

On admission the patient had a mild fever of 38.5°C but the other vital signs were normal. Clinical examination was unremarkable except for a moderately enlarged liver and oral ulcers. In addition to the oral lesions, multiple painless, hard-edged ulcers and oedematous swelling were found on the penis and scrotum (Figure 2, upper right image). No anal lesions were present. Signs of fluid overload such as peripheral oedema were absent. No abnormalities were found upon neurological examination, there being no signs of meningismus.

Serum laboratory values on admission showed increased inflammation markers C-Reactive Protein (CRP) and procalcitonin, moderately elevated liver enzymes, moderate hypoalbuminemia and severe hyponatremia (Table 1). Moreover markedly reduced levels of Thyroid Stimulating Hormone (TSH), thyroxine (T4) and triiodothyronine (T3) were noted, indicative of a centrally-induced hypothyroidism. Creatinine and uric acid levels were subnormal. Urine analysis revealed high osmolality and sodium, consistent with the Syndrome of Inappropriate Antidiuretic Hormone Secretion (SIADH).

**Table 1:** Serum and urine laboratory values at presentation.

Plasma	Result	Normal Range	Unit
Sodium	113	135-145	mmol/l

Potassium	4.4	3.5-5	mmol/l

Calcium	1.8	2-2.6	mmol/l

Chloride	87	97-108	mmol/l

Phophate	0.6	0.8-1.6	mmol/l

Osmolality	240	280-300	mosm/kg

Uric acid	2.9	3.4-7 l	mg/d

Albumin	27	35-50	g/l

GOT	75	10-50	U/I

GPT	46	10-50	U/I

GGT	166	10-66	U/I

CRP	160	< 1	mg/l

Procalcitonin	2.8	< 0.5	ng/l

TSH	0.07	0.4-2.5	mU/l

fT3	0.2	0.8-1.8	ng/dl

fT4	1.5	2.2-4.5	pg/ml

Spontaneous urine specimen			

Protein	negative		

Blood	negative		

Specific gravity	1.015	1.01-1,03	g/ml

24-hour urine specimen			

Urine osmolality	540	50-1400	mosm/kg

Urine sodium	30		mmol/l

Urine volume	1200		Ml

Cerebro-spinal fluid analysis			

White blood cell count	6	< 4	/μl

Red blood cell count	< 1	< 1	/μl

Protein	0.59	< 0.45	g/l

Laboratory diagnosis of syphilis in this patient included serology and a Real-Time Polymerase Chain Reaction (RT-PCR) assay against the *polA *gene of *Treponema pallidum *[6]. Serologic tests revealed a reactive Rapid Plasma Reagin (RPR)-titer of 1:32, a *T. pallidum *haemagglutination assay (TPHA) titer of 1:2560 and positive *T*. *pallidum *IgM and IgG immunoblots (recomBlot, Mikrogen, Germany). The RT-PCR detected the presence of *T. pallidum *DNA in the swabs from oral and genital lesions, but not from the anal swab. All other bacterial, fungal and mycobacterial cultures of this patient were negative. Surrogate markers of the HIV infection showed a viral load of 2 × 10^5 ^c/ml and a CD4 count of 191/μl. Additional virological studies revealed chronic HBV and HCV infection with viral loads of 55 c/ml (12 IU/ml HBV-s-NAT) and 10^7 ^IU/ml (HCV-NAT).

CSF analysis indicated increased blood-brain-barrier permeability with an elevated white blood cell count of 6/μl (normal <4/μl) and protein level of 0.59 g/l (normal <0.45 g/l). No blood contamination was detected (red blood cell count <1/μl). The *T*. *pallidum *IgG immunoblot from the cerebrospinal fluid was also positive showing the presence of additional and higher-intensity bands compared to the serum sample. This indicated intrathecal antibody production and suggested neurosyphilis. HIV load in CSF was 2 × 10^4 ^c/ml, while PCR based testing for JC Virus (JCV), Herpes Simplex Virus 1/2 (HSV 1/2), Varicella Zoster Virus (VZV) and Cytomegalovirus (CMV) were negative.

Sonography found a normal thyroid size and structure, however, a discrete reduction of the perfusion was noted. A computed tomography (CT) scan showed generalized lymphadenopathy, moderate enlargement of liver and spleen, and extension of the genital oedema to the lower abdominal wall (Figure 2, lower image). Cerebral Magnetic Resonance Imaging (MRI) including pituitary imaging showed no abnormalities apart from sinusitis. Of note, no signs of elevated intracranial pressure were detected. CSF pressure was not measured during lumbar puncture.

The patient was treated with intravenous ceftriaxone 2 g/d for 14 days due to a reported allergy to penicillin. Prednisolone at 50 mg/d (tapering scheme over 4 weeks) was added on the recommendation of the ophtalmologist in order to treat papillitis. Fluconazole was included for oesophageal thrush, and trimethoprim-sulfomethoxazole for pneumocystis prophylaxis. Antiretroviral Therapy (ART) was started after completion of ceftriaxone. Because of the high viral load in the CSF, a regimen with a high CNS Penetration-Effectiveness rank was chosen, consisting of Lopinavir/Ritonavir (LPV/r), Tenofovir (TDF), Lamivudine (3TC), Zidovudine (AZT) [7].

**Figure 3:**
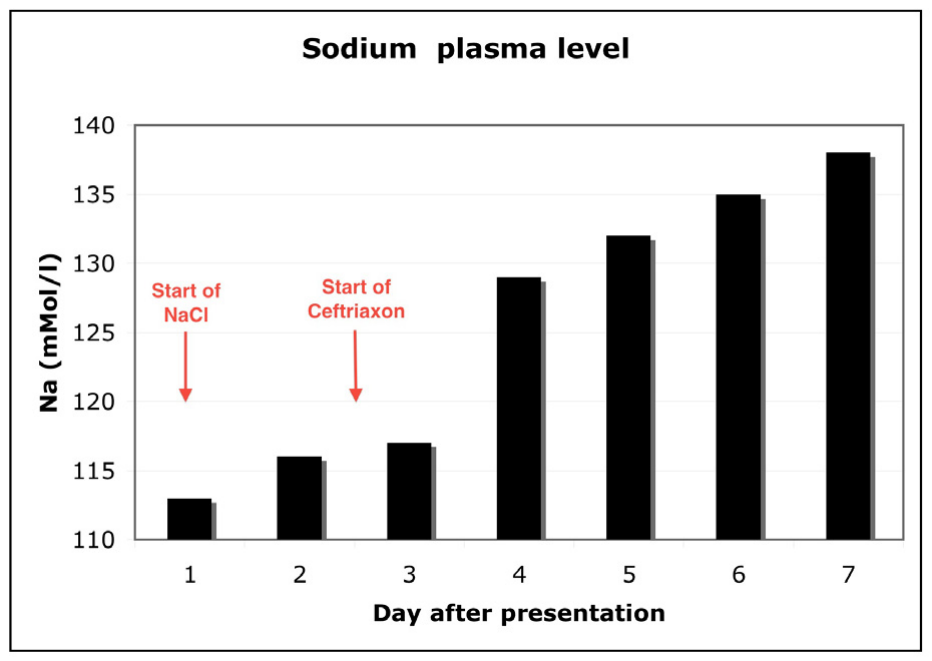
**Plasma sodium concentration**. On the day of presentation oral sodium chloride substitution and water restriction were started. Sodium concentration normalized rapidly after the start of ceftriaxone treatment while sodium chloride substitution was not increased.

A therapeutic response to syphilis treatment was observed with normalization of sodium levels (Figure 3), healing of oral ulcers and improvement of the patient's general condition within one week. TSH levels normalized within 3 weeks without any additional specific thyroid treatment, whereas T4 and T3 remained low for longer (Table 2). Visual acuity also improved markedly from 0.02 to 0.2 and papillitis was no longer present after 3 weeks of treatment (Figure 1, lower image).

**Table 2:** Time course of thyroid values

Days after presentation	TSH [0.4-2.5 mU/l]	fT3 [0.8-1.8 ng/dl]	fT4 [2.2-4.5 pg/ml]
1	0.07	0.2	1.5

5	0.15	0.2	1.3

7	0.22	0.3	1.3

9	0.38	Not measured	Not measured

20	1.44	0.6	1.9

## Discussion

Following the identification of painless ulcers on the genitalia and mouth, the clinical diagnosis of syphilis was straightforward, although the patient had no complaints apart from visual impairment initially denying any risk factors. Interestingly, while multiple primary lesions were still present the patient also had generalized lymphadenopathy, neurosyphilis and ocular syphilis, which are characteristic of secondary syphilis. An overlap of primary and secondary syphilis occurs in 75% of HIV co-infected patients [8]. The rate of ocular and neurological involvement is higher in patients with HIV infection [9,10] and a CD4 count of <350/μl is associated with an increased risk of neurosyphilis [11].

We retained the diagnosis of early syphilis with ocular and CNS involvement upon clinical symptoms and microbiological findings in serum and CSF. Remarkably, CNS involvement possibly led to SIADH. This has not previously been described in neurosyphilis, as far as PubMed searches are concerned, for "neurosyphilis AND hyponatremia" or "neurosyphilis AND SIADH". Although hypothalamic and pituitary function were greatly altered, no structural changes could be found on the MRI.

Differential diagnosis of hyponatremia was considered in detail, especially cerebral salt-wasting syndrome, syphilis nephritis, hypothyroidism and adrenal insufficiency. The characteristics of cerebral salt-wasting syndrome are dehydration and polyuria [12] and were therefore excluded by clinical diagnosis. Syphilis nephritis is a membranous glomerulonephritis characterized by proteinuria and potentially microscopic haematuria with impaired renal function [13,14]; none of which were found in the patient (Table 1). Primary adrenal insufficiency can cause hyponatremia by lack of mineralocorticoid secretion, leading to urinary sodium wasting, hyperkalemia and dehydration. Secondary adrenal insufficiency also causes an inappropriate antidiuresis by ADH secretion [15]. Potential involvement of adrenal dysfunction in the pathology of the presented patient cannot be excluded, because an Adrenocorticotropic Hormone (ACTH) stimulation test was not performed.

Hypothyroidism can also be a cause of hyponatremia, although some authors doubt a causal relationship [16]. Two possible mechanisms have been described. Hypothyroidism is thought to induce SIADH [17,18], or lead to impaired renal function because of decreased cardiac output [19]; however, renal function in our patient was normal.

The absence of dehydration, normal potassium levels, and the elevated urine osmolality and urine sodium were highly suggestive of SIADH. Whether SIADH in this patient occurred secondary to hypothyroidism or "directly" by neurosyphilis cannot be conclusively determined. Previous descriptions of hyponatremia as a consequence of hypothyroidism report a milder decrease of sodium (> 120 mMol/l) and a urine osmolality of <500 mosm/kg [16]. Furthermore, the anatomical proximity of the affected structures in the hypothalamic-pituitary region suggests that neurosyphilis might have played a direct role.

The combination of decreased TSH and decreased thyroid hormones T3/T4 points to a centrally-induced hypothyroidism [20]. Other infectious causes of central hypothyroidism have been described, such as toxoplasmosis [21] and CMV-encephalitis [22], especially in HIV-positive patients [23], but not in neurosyphilis. According to serology and CSF analyses none of these infections were present in the patient. In one case HIV itself was thought to be the cause of central hypothyroidism [24], as improvement occurred under AZT treatment alone. In our case, it is unlikely that the virus itself was causative since remission occurred rapidly under treatment for syphilis alone, even though HIV could be detected in the CSF. ART was initiated after completion of ceftriaxone treatment. No signs of HIV encephalopathy were seen on the MRI.

In recent years non-thyroidal illness syndrome (NTIS) has been recognized as a cause of central hypothyroidism. NTIS has been described in malnourished and critically ill patients due to decreased leptin levels, leading to a diminution in hypothalamic Thyreotropin Releasing Hormone (TRH) [20]. As the patient exhibited weight loss, this differential diagnosis had to be considered. The rapidity of the therapeutic response might suggest involvement of *Treponema pallidum *in the pathogenesis, but the complexity of the presented case makes it difficult to appoint one single cause.

The incidence of syphilis is rising again worldwide [1], in Germany it is currently 3.87 cases per 100000 persons [25]. Disease rates in some countries of Eastern Europe are 10 times higher [26]. Therefore, syphilis should always be included as a differential diagnosis not only in the infectious disease department, but also in other specialties, especially neurology, psychiatry, dermatology and ophthalmology.

Primary diagnosis of syphilis by ophthalmologists has been repeatedly reported in recent years [27-29]. In some cases antibiotic treatment alone was used. As papillitis was also present in our case, we chose to add prednisolone to the treatment. A considerable improvement of visual acuity and resolution of papillitis occured within 3 weeks (Figure 1).

Another notable feature of this case was the diagnosis of syphilis, HIV, and chronic hepatitis B and C co-infection at the same time. The exact time-course of the infections cannot be elucidated. Increasing incidence of co-infection of syphilis and HIV has been found in epidemiological studies [25,30]. The reasons for this concomitance are shared risk factors especially, as in this case, MSM, but also due to increased transmission of HIV, HBV and HCV in the presence of syphilitic lesions [25,31].

## Conclusion

The case presented here suggests that SIADH and potentially central hypothyroidism should be added to the list of symptoms of neurosyphilis. Even today, previously unknown presentations of neurosyphilis arise, especially in HIV infected patients [32]. HIV co-infection leads to a more rapid and severe course of the disease, and broadens the differential diagnosis for CNS pathologies.

Clinicians of all specialties should be aware of syphilis as a differential diagnosis in these times of increasing incidence.

## Competing interests

The authors declare that they have no competing interests.

## Authors' contributions

KM contributed to diagnosis and treatment of the patient as physician and wrote the manuscript, VF contributed to diagnosis and treatment as physician, AK carried out the microbiological studies and helped to draft the manuscript, TD contributed to diagnosis and treatment as attending physician and did the outpatient follow-up, JL contributed to diagnosis and treatment as attending professor and revised the manuscript.

All of the authors have given final approval of the version to be published.

## Pre-publication history

The pre-publication history for this paper can be accessed here:

http://www.biomedcentral.com/1471-2334/11/17/prepub
